# Synthesis and design of Ag–Fe bimetallic nanoparticles as antimicrobial synergistic combination therapies against clinically relevant pathogens

**DOI:** 10.1038/s41598-021-84768-8

**Published:** 2021-03-05

**Authors:** A. L. Padilla-Cruz, J. A. Garza-Cervantes, X. G. Vasto-Anzaldo, Gerardo García-Rivas, A. León-Buitimea, J. R. Morones-Ramírez

**Affiliations:** 1 Universidad Autónoma de Nuevo León, UANL, Facultad de Ciencias Químicas, Av. Universidad S/N. CD. Universitaria, San Nicolás de Los Garza, NL 66455 Mexico; 2 Centro de Investigación en Biotecnología y Nanotecnología, Facultad de Ciencias Químicas, Universidad Autónoma de Nuevo León, Parque de Investigación E Innovación Tecnológica, Km. 10 autopista al Aeropuerto Internacional Mariano Escobedo, Apodaca, Nuevo León 66629 México; 3 Cátedra de Cardiología Y Medicina Vascular, Escuela de Medicina. Tecnologico de Monterrey, Monterrey, Nuevo León Mexico; 4 Centro de Investigación Biomédica, Hospital Zambrano-Hellion, Tecnologico de Monterrey, San Pedro Garza-García, Nuevo León Mexico

**Keywords:** Antibiotics, Antimicrobial resistance, Nanoparticles

## Abstract

The inappropriate use of antibiotics and the inadequate control of infections have led to the emergence of drug-resistant strains. In recent years, metallo-pharmaceutics and metallic nanoparticles have been proposed as potential alternative antimicrobials due to their broad-spectrum antimicrobial properties. Moreover, recent findings have shown that combinations of transition metal compounds can exhibit synergistic antimicrobial properties. Therefore, the synthesis and design of bimetallic nanoparticles is a field worth exploring to harness the interactions between groups of metals and organic complex structures found in different microbial targets, towards the development of more efficient combinatorial antimicrobials composed of synergistic metals. In this study, we present a green synthesis of Ag–Fe bimetallic nanoparticles using an aqueous extract from the leaves of *Gardenia jasminoides*. The characterization of the nanoparticles demonstrated that the synthesis methodology produces homogenously distributed core–shell Ag–Fe structures with spherical shapes and average diameter sizes of 13 nm (± 6.3 nm). The Ag–Fe bimetallic nanoparticles showed magnetic and antimicrobial properties; the latter were evaluated against six different, clinically relevant multi-drug-resistant microbial strains. The Ag–Fe bimetallic nanoparticles exhibited an antimicrobial (bactericidal) synergistic effect between the two metals composing the bimetallic nanoparticles compared to the effects of the mono-metallic nanoparticles against yeast and both Gram-positive and Gram-negative multidrug-resistant bacteria. Our results provide insight towards the design of bimetallic nanoparticles, synthesized through green chemistry methodologies, to develop synergistic combinatorial antimicrobials with possible applications in both industrial processes and the treatment of infections caused by clinically relevant drug-resistant strains.

## Introduction

The fast spread of drug-resistant infectious diseases poses a global health threat^1^. If the trend continues at the current speed, by 2050, it will lead to 10 million people dying every year from drug-resistant infections^2^. Metal nanoparticles (NPs) have great potential to be utilized as antimicrobial agents^3^. Noble metal NPs, such as silver (Ag) and gold (Au), have been shown to exhibit strong and sustainable antibacterial action against a wide array of microorganisms; therefore, they have been utilized in medical devices, food preservatives, dental resin composites, cosmetics, medical device coatings, implants, and medical instruments^4^. Bimetallic NPs have gained specific attention in the last decade due to their optical, electronic, magnetic, and catalytic properties, which, in most cases, are significantly distinct from their monometallic counterparts^4^. Bimetallic NPs are synthesized by combining two different metal elements, resulting in various morphologies and structures^5^.

Green synthesis provides a new scope for NP synthesis since it is an eco-friendly, simple, stable, rapid, and less-costly method^6^. In general, the synthesis of bimetallic NPs involves the mixing of two different aqueous metal solutions with an environmentally friendly reducing agent, such as a plant extract^7^. The plant phytochemicals, with antioxidant or reducing properties, are believed to reduce metal ions into metal nanoparticles^8^. Theoretically, metal ions with a stronger reduction potential are reduced faster than metal ions with a weaker reduction potential. Such is the well-known system of the Au–Ag bimetallic NPs, where Au ions are reduced first, forming the nuclei, while Ag ions are reduced later and adsorbed onto the Au particles, forming a core–shell structure^9^.

*Gardenia jasminoides* (*G*. *jasminoides*), an evergreen tree, is commonly used in traditional Chinese medicine due to its multiple biological activities (antioxidant properties, hypoglycemic effect, and inhibition of inflammation)^10^. It has been reported that *G*. *jasminoides* leaves possess alkaloid, flavonoid, saponin, tannin, and phenolic compounds^11^. Therefore, extracts from the leaves of *G*. *jasminoides* possess a high reducing potential for the synthesis of metallic nanoparticles such as palladium, iron, and silver^12–14^.

The synthesis of bimetallic nanoparticles using plant extracts is a promising method since it has proved to be cheaper, safer, simpler, quicker, and easier than conventional methods^8^. The green synthesis of Au–Ag bimetallic nanoparticles and their biomedical applications have been extensively reported^15–18^. On the other hand, silver-iron (Ag–Fe) bimetallic nanoparticles have several applications in optical, medical, and the remediation fields^19–22^. However, there are synthesis methods and important properties and characteristics that still need to be investigated.

Therefore, in this study we synthesized Ag, Fe, and Ag–Fe bimetallic nanoparticles using *G*. *jasminoides* extract as a reducing agent in the redox synthesis of the nanomaterials. We characterized the nanostructures by spectroscopic and microscopic analyses and studied their magnetic properties as well as the minimal inhibitory concentration (MIC) and the minimum bactericidal and fungicidal concentration (MBC and MFC) against clinically relevant pathogenic strains. The findings and results presented in this work contribute to the advancement of knowledge on synthesis and antimicrobial properties of mono and bimetallic nanoparticles composed of Ag and Fe. We demonstrated the ability to use green synthesis methods to design and synthesize magnetic bimetallic nanoparticles composed of Ag and Fe and demonstrated that they exhibit a synergistic antimicrobial effect. Moreover, the results here described give insight towards the development of novel and more effective antimicrobial therapies for different applications and in the possible treatment of infections caused by both sensitive and drug-resistant clinically relevant pathogens.

## Results and discussion

### Nanoparticles characterization

Nanomaterials, including bimetallic nanoparticles, are of growing interest in many applications. Bimetallic nanoparticles can exhibit a variety of properties due to the synergistic effect created when two different metals are combined. This phenomena can enhance their features and properties and therefore expand or accentuate their applications as antimicrobial agents, drug delivery systems, and as imaging agents^23^. Nonetheless, while much research has been performed on monometallic nanoparticles, fewer studies have explored the use of bimetallic nanoparticles in those fields. We therefore first explored the synthesis of Ag, Fe, and Ag–Fe bimetallic NPs by monitoring the color changes in the reaction mixture, followed by their physical–chemical characterization and their antimicrobial properties using various analytical techniques.

**Figure 1:**
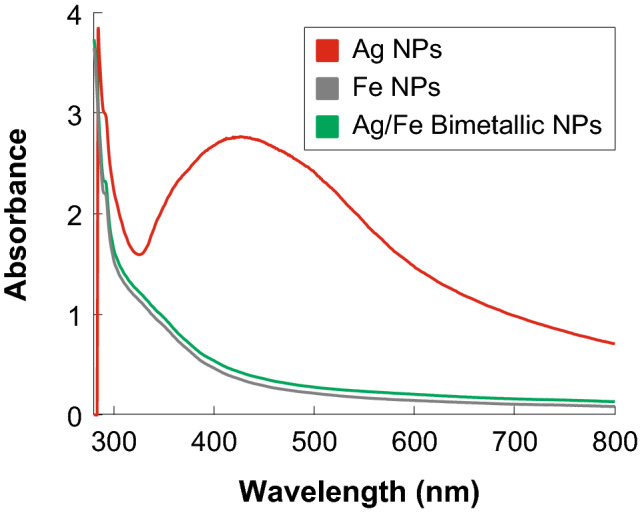
UV–visible absorbance spectra of silver (Ag), iron (Fe) nanoparticles, and Ag–Fe bimetallic nanoparticles synthesized using an aqueous extract from leaves of *Gardenia jasminoides*.

For the synthesized Ag-NPs, a wide absorption band can be observed between 380 and 580 nm with a maximum absorption peak at 425 nm (Fig. 1). This result agrees with previous reports where the peak corresponding to silver nanoparticles is well documented to be between 390 and 580 nm^24^. For the case of the Fe-NPs and the synthesis of Ag–Fe bimetallic nanoparticles, both showed the characteristic UV–Vis spectrum, with a peak at 290 nm and a slight peak at 350 nm, corresponding to metallic iron (Fig. 1). These peaks are related to Fe residues and the Fe surface plasmons' collective oscillation. These absorption bands of surface resonance plasmon have been reported to be present in iron nanoparticles^25^. As can be seen, a characteristic UV–Vis absorption spectrum was displayed when the silver and iron nanoparticles were analyzed independently. However, for the bimetallic nanoparticles, an absorption spectrum resembling that of iron nanoparticles was observed, suggesting the formation of silver-iron core–shell bimetallic nanoparticles. Similar results have been observed in the synthesis of diverse bimetallic nanoparticles^26, 27^.

**Figure 2:**
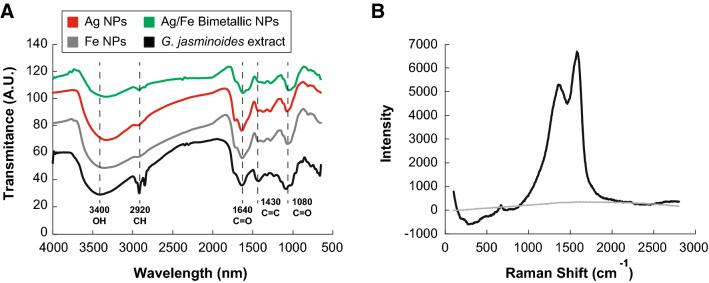
Fourier Transform Infrared (FT-IR) spectra (**A**) of silver (Ag), iron (Fe) nanoparticles, and Ag-Fe bimetallic nanoparticles synthesized using an aqueous extract from leaves of *Gardenia jasminoides* and the RAMAN spectra of the (**B**) aqueous extract from leaves of *Gardenia jasminoides*.

To identify the biomolecules contained in the *G. jasminoides* leaves extract, responsible for the reduction of Ag^+^ and Fe^+^ ions in the synthesis of nanoparticles, an FT-IR analysis was performed (Fig. 2A). We identified IR bands centered at 3400, 2920, 1640, 1430, and 1080 cm^−1^. The band at the 3600–3000 cm-1 region corresponds to hydroxy groups stretching (O–H). The band at 2950 cm^−1^ corresponds to the stretching of aliphatic (C–H). The bands at the 1640 and 1430 cm^−1^ region can be assigned to C=O and C=C stretching, respectively. Finally, the 1080 cm-1 band appears due to the stretching of C-O. The FT-IR spectra of the *G. jasminoides* extract resembled those previously reported^12^. After the synthesis of the Ag, Fe, and Ag–Fe bimetallic nanoparticles with the extract, the absorption peaks appeared weaker than the peaks previous to the reaction synthesis. This result indicates that the extract coated the nanoparticles, reduced metallic ions, and stabilized the nanoparticles during the synthesis. According to several authors, these reducing and stabilizing properties can be attributed to polyphenolic compounds like flavonoids and phenolic acids present in the *G. jasminoides* extract^10, 13, 28^. In addition, the RAMAN spectra in Fig. 2B confirmed the presence of two absorption peaks between 1300 and 1500 cm^−1^, which are characteristic to aromatic rings related to polyphenolic compounds^29^.

**Figure 3:**
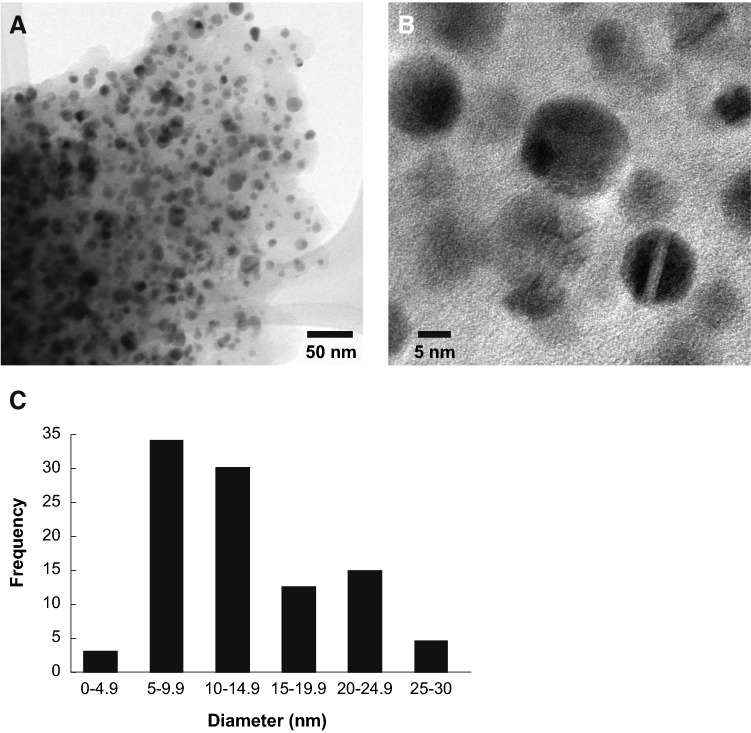
Transmission Electron Microscopy (TEM) characterization of Ag–Fe bimetallic nanoparticles synthesized using an aqueous extract from leaves of *Gardenia jasminoides* (**A**) Low magnitude TEM (**B**) High-resolution TEM images and (**C**) Size distribution histogram of Ag–Fe bimetallic nanoparticles.

The composition, particle size, and size distribution of the Ag–Fe bimetallic NPs were studied by several methods. TEM analysis (Fig. 3A) confirmed that the bimetallic nanoparticles displayed a spherical morphology, a homogenous distribution with particles that ranged from 3 to 30 nm in diameter and an average diameter size of 13 nm with an SD + /− 6.13 nm (Fig. 3B and C). The Ag–Fe bimetallic nanoparticles obtained in this study were smaller than those previously reported, where their average size ranged from 20 to 60 nm^30, 31^. However, the differences are related to the reducing and stabilizing agents used during the synthesis process^32^. The high-resolution TEM image displayed in Fig. 3B shows clear fringes and morphology that suggests the formation of a core–shell (Ag-core Fe-shell) nano-arrangement of the bimetallic nanoparticles, as reported on the literature^30, 33^.

**Figure 4:**
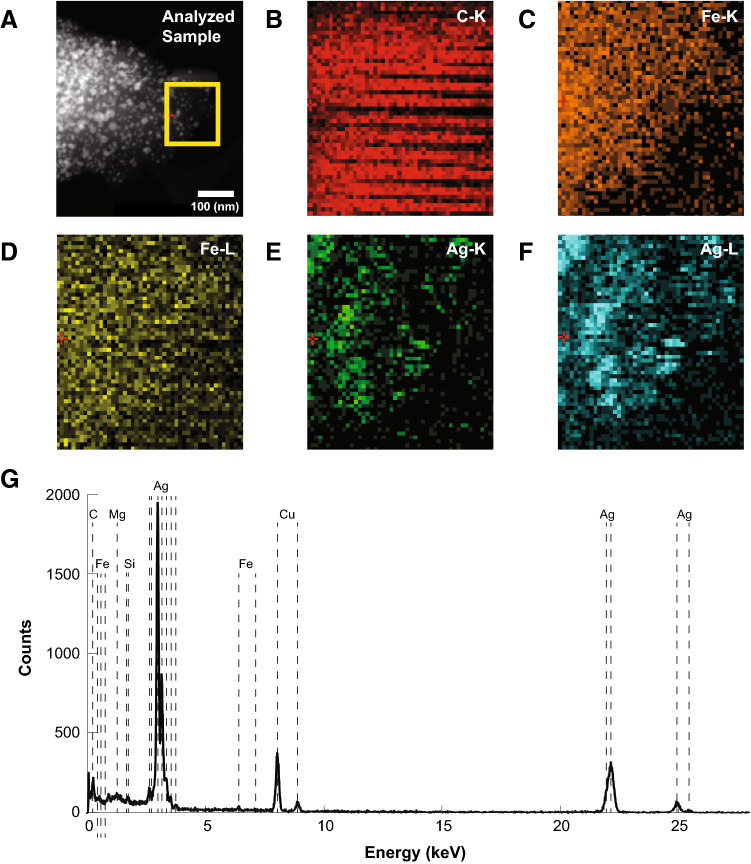
Darkfield image (**A**)–(**F**) and energy dispersive X-Ray (EDX) spectra (G) of Ag–Fe bimetallic nanoparticles synthesized using an aqueous extract from leaves of *Gardenia jasminoides*.

The presence of silver and iron as components of the Ag–Fe bimetallic nanoparticles were confirmed through a dark-field microscopy analysis (Fig. 4A–F). The results showed a clear overlap of the Fe and Ag within the selected, analyzed field, corroborating the bimetallic nanoparticle formation. In addition, carbon (C) was also analyzed to detect the element present in the reducing organic extract. Further analysis using energy dispersive X-Ray (EDX) spectroscopy was carried out to obtain an elemental spectrum in the Ag–Fe bimetallic nanoparticle sample. Figure 4G shows the EDX spectra of the synthesized Ag–Fe bimetallic NPs, where the binding energies of metallic silver are observed, at approximately 3 keV, while the peaks around 1, 6, and 7 keV are related to the binding energies of Fe^34, 35^. The elemental composition of the bimetallic nanoparticles was determined by EDX and confirmed that the Ag/Fe bimetallic NPs were composed of 58.59% Ag and 41.4% Fe.

### Magnetic properties

**Figure 5:**
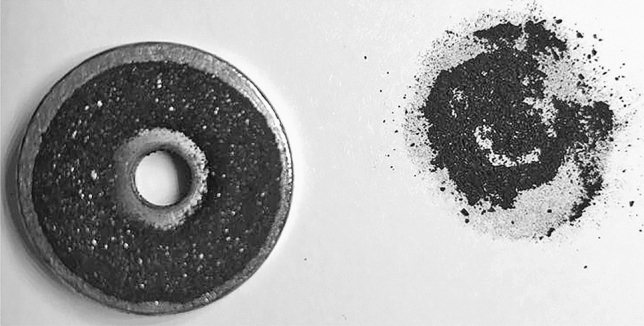
Magnetic property of Ag–Fe bimetallic nanoparticles synthesized using an aqueous extract from leaves of *Gardenia jasminoides*.

Magnetic NPs have been reported as non-immunogenic nanomaterials with excellent biocompatibility, suitable for medical applications^36^. Therefore, the magnetic bimetallic nanoparticles are an outstanding option for diagnostic (sensing and imaging) and therapeutic applications (thermal treatments and drug-delivery systems)^37^. Hence, in the present study, we evaluated the magnetic properties of the Ag–Fe bimetallic NPs (Fig. 5). As can be observed, when an external magnetic field is applied to the Ag–Fe bimetallic NPs, they adhered to the magnet, therefore exhibiting magnetic properties. This magnetic property has previously been described in nanoparticles containing iron^30, 31^.

### Antimicrobial activity

The emerging resistance in bacteria and the high costs of advanced antimicrobial drugs have encouraged scientists to search for effective, economically viable, and broadly applicable drugs^38^. In search of a new therapeutic alternative, we determined the minimal inhibitory concentrations (MIC) of AgNPs, FeNPs, and Ag–Fe bimetallic NPs against Gram-positive, Gram-negative bacteria, and a yeast using the broth microdilution method (Table 1).

**Table 1:** Minimal Inhibitory Concentration (MIC), S value, Minimum Bactericidal Concentration (MBC) and Minimum Fungicidal Concentration (MFC) of silver nanoparticles (AgNPs), iron nanoparticles (FeNPs), Ag-Fe bimetallic nanoparticles, and combination of AgNPs + FeNPs, prepared using *Gardenia jasminoides* leaves aqueous extract against clinically relevant pathogens.

Strain	MIC AgNPs (ppm)	MIC FeNPs (ppm)	MIC Bimetallic NPs (Ag / Fe*) (ppm)	MIC AgNPs + FeNPs (Ag / Fe*) (ppm)	S value	MBC or MFC of Bimetallic NPs (ppm)
*S. aureus* ATCC 6538	125	> 250	125 (73.23/51.75)	125 (73.23/51.75)	0.53	250
*S. aureus* ATCC 29,213	125	> 250	125 (73.23/51.75)	125 (73.23/51.75)	0.40	250
*S. aureus* Multidrug-resistant	125	> 250	250 (146/103.5)	250 (146/103.5)	0.80	500
*P. aeruginosa* ATCC 27,853	62.5	> 250	31.25 (18.3/12.9)	31.25 (18.3/12.9)	0.89	62.5
*P. aeruginosa* ATCC DMS 50,071	15.62	> 250	31.25 (18.3/12.9)	31.25 (18.3/12.9)	0.80	62.5
*P. aeruginosa* Multidrug-resistant	31.25	> 250	15.62 (9.15/6.42)	15.62 (9.15/6.42)	0.90	31.25
*E. coli* ATCC 11,229	125	> 250	125 (73.23/51.75)	62.5 (36.6/25.9)	0.40	250
*C. albicans* Clinical isolate	125	> 250	62.5 (36.6/25.9)	62.5 (36.6/25.9)	0.08	125

*The elemental composition of Ag and Fe was considered to analyze nanoparticles' antimicrobial activity (bimetallic and AgNPs + FeNPs): 58.59% of Ag and 41.4% of Fe.

S value: S > 0 Synergistic; S = 0 Additive; S < 0 Antagonistic.

The present study showed that AgNPs had an antimicrobial effect in concentrations ranging from 15.62 to125 ppm. The MIC values of *Staphylococcus aureus* (*S*. *aureus*), a Gram-positive bacteria, were the same in the ATCC and the multidrug-resistant strains (125 ppm). In contrast, *Pseudomonas aeruginosa* (*P*. *aeruginosa*), a Gram-negative bacteria, exhibited lower MIC values (from 15.62 to 62.5 ppm) in both ATCC and multidrug-resistant strains, compared to the Gram-positive bacteria. *Escherichia coli* (*E. coli*; Gram-negative bacteria) and *Candida albicans* (*C*. *albicans*; yeast), displayed MIC values equal to the Gram-positive bacteria, *S. aureus* (125 ppm). These trends in MIC results are in agreement with previous studies^39–41^.

The MIC results of the present study demonstrated that the FeNPs exhibited no antimicrobial activity (> 250 ppm), at the explored ranges, in all tested strains. As we previously mentioned, the antibacterial activity of FeNPs has not been thoroughly studied. Most of the studies have evaluated this activity by the well-diffusion method in Gram-positive and Gram-negative bacteria^13, 42, 43^. We studied the antimicrobial activity using the broth microdilution method; therefore, the results are difficult to comparable.

On the other hand, the MIC values of Ag–Fe bimetallic NPs were similar or lower than those obtained with AgNPs and FeNPs alone. We considered the elemental composition of Ag and Fe in the bimetallic NPs to analyze their antimicrobial activity (58.59% of Ag and 41.4% of Fe). In general, the MIC values for Ag–Fe bimetallic NPs in *S*. *aureus* (*S. aureus* ATCC 6538, *S. aureus* ATCC 29213) were comparable to those obtained with AgNPs (125 ppm). However, the elemental composition demonstrated that silver and iron concentrations were 73.23 and 51.75 ppm, respectively. Interestingly, the antibacterial effect of Ag–Fe bimetallic NPs was more evident against *P*. *aeruginosa*. As shown in Table 1, the MIC value in *P*. *aeruginosa* ATCC 27853 and ATCC DMS 50071 was 31.25 ppm, while the multidrug-resistant strain was 15.62 ppm. When we analyzed the elemental composition, it was observed that the concentration of silver and iron was reduced by 3.4 and 19-fold, respectively, compared to AgNPs and FeNPs MICs values. These data show the potential effects of Ag–Fe bimetallic NPs in multidrug-resistant bacteria and suggests the importance of the synergistic interactions of these bimetallic NPs, especially for other Gram-negative bacteria with different antibiotic resistance profiles.

Regarding the antifungal activity of Ag–Fe bimetallic NPs in *C. albicans* (clinical isolate), we observed a MIC value of 62.5 ppm (36.6 and 25.9 ppm, Ag and Fe, respectively), which was reduced by 3.4 and 9.6-fold, compared to AgNPs and FeNPs MICs values. Moreover, it is worth highlighting that the MICs reported in this study were found to be lower than those previously reported for Ag–Fe bimetallic NPs in *S. aureus* ATCC, *E. coli* ATCC*, P. aeruginosa* ATCC, and multidrug-resistant *P. aeruginosa*^44, 45^.

The synergistic antimicrobial activity of Ag–Fe bimetallic NPs in a complex matrix, such as bacterial cultures, could follow a non-linear dosage-response behavior. This kind of response is better analyzed when using a synergy test (such as the Bliss independence model) that takes into account the effect of the antimicrobial agent on the bacterial culture, rather than tests that assume all types of antimicrobial agents and their combinations have a linear dosage-respond curve, like the isobologram and the FIC index interpretations^46, 47^. We therefore evaluated whether the synergistic effect, measured by the Bliss Independence Model, observed in Ag–Fe bimetallic nanoparticles was conserved or modified after the combination of individually synthesized AgNPs and FeNPs. The results demonstrate that all of the interactions in the bimetallic nanoparticles were found to be synergistic (Table 1). Moreover, the combination of AgNPs + FeNPs was also found to be synergistic in the antimicrobial efficacy against *E. coli* ATCC 11229. In addition, it is critical to keep in mind that one of the main advantages of using bimetallic nanoparticles instead of single metal nanoparticles is that incorporating two individual metals in a single entity makes their synthesis a greener procedure and their delivery a much simpler process^15^. Moreover, this process significantly reduces the quantities of hazardous wastes produced during the biosynthesis of nanoparticles^48^.

The antimicrobial action of Ag–Fe bimetallic NPs seems to depend on the differences in the cell walls or cell membrane composition of the microorganism. However, the antimicrobial mechanism of Ag–Fe bimetallic nanoparticles has not been elucidated yet. Nonetheless, core–shell nanostructures display a more significant effect than that observed in monometallic nanoparticles^44^. Porins could act as a transporter channel for the nanoparticles to and from the cell^49^. It is also known that silver nanoparticles can anchor to the bacterial cell wall and penetrate it. Once inside, they contribute to the formation of free radicals generating intracellular oxidative stress, which leads to cell death^50^. Recently, it has been described that iron might interact with specific amino acids (–SH groups of cysteine) present in proteins of the bacteria cell wall. Particularly, cysteine's thiol side chain has been identified as the most susceptible to gain electrons from the oxidizing species^21^. Therefore, we propose that the antimicrobial mechanism of Ag–Fe bimetallic nanoparticles is carried out in two steps: first, iron, which is coating the nanoparticles, oxidizes the thiol side chain in cysteine leading to changes in the primary structure of proteins, alteration in bacterial cell wall permeability and finally cell death. Second, due to the altered permeability of the cell wall, many nanoparticles are uptake by the bacteria. Silver ions are then released from NPs to the cytoplasm inducing oxidative stress, causing DNA alterations and disruption of membrane morphology. Thus, silver and iron contribute to disrupting cellular structures, intracellular biological functions, and lead cell death. Nonetheless, further studies are needed to support this hypothesis.

Next, we determined the minimum bactericidal and fungicidal concentration of Ag–Fe bimetallic NPs to demonstrate their ability to kill bacteria and yeast (Table 1). A bactericidal agent is defined as a material with a ratio of MBC to MIC ≤ 4. Antibiotics with a ratio of MBC to MIC > 4 are defined as bacteriostatic agents^51^. The bactericidal agent kills bacteria rapidly and reduces bacterial resistance development; hence they are a better choice for clinicians than bacteriostatic agents^52^. Our results showed that the MBC and MFC values were twice the MIC concentrations; thus, the Ag–Fe bimetallic nanoparticles were bactericidal and fungicidal.

Finally, we conducted a bibliographic search on the evaluation of the biocompatibility of the Ag–Fe bimetallic nanoparticles. A recent study demonstrated that hybrids silver-iron nanoparticles significantly enhanced the cytotoxicity of MCF-7 breast cancer cells. Therefore, these nanoparticles could be a potent anticancer drug^53^. Another study evaluated magnetite/silver nanoparticles biocompatibility in Vero cells (Kidney epithelial cells). The results evidenced low cytotoxicity, hemolytic, and platelet aggregation tendency, which confirm a high biocompatibility^54^. Moreover, a cytotoxicity study of iron-silver core–shell nanoparticles (FeO/AgNPs) was carried out on Vero cell line, and the results showed that these nanoparticles were biocompatible up to 500 µgml^−1^ concentration. Hence, even though further studies are required on our specific Ag–Fe bimetallic nanoparticles system, these studies suggest the biocompatibility of our Ag–Fe bimetallic nanoparticles and hint for their potential as biocompatible nanomaterials with biological and biomedical applications.

## Conclusions

The green synthesis of bimetallic nanoparticles using natural extracts is gaining research attention for environmental and medical applications. In the present study, silver nanoparticles (AgNPs), iron nanoparticles (FeNPs), Ag–Fe bimetallic nanoparticles, and the combination of AgNPs + FeNPs, were prepared in a redox reaction using the aqueous *G*. *jasminoides* leaves extract as a reducing agent. The evaluation of the antimicrobial activity of bimetallic NPs showed synergistic bactericidal and fungicidal effects against Gram-positive and Gram-negative bacteria and yeast. Our results demonstrate the ability to use green synthesis methods to design and synthesize magnetic bimetallic nanoparticles composed of metals that synergize their antimicrobial effects. Moreover, this work gives insight into the development of novel and more effective antimicrobial agents with potential as bactericidal and fungicidal agents, to treat drug-resistant infections, and in other biomedical applications.

## Methods

### Microbial strains and culture conditions

Strains used in this study include *S. aureus* ATCC 6538, *S. aureus* ATCC 29213, *E. coli* ATCC 11229, *P. aeruginosa* ATCC 27853, and *P. aeruginosa* DSM 50071. Meanwhile, clinical isolates from a Gram-negative multidrug-resistant strain of *P. aeruginosa* (kanamycin, chloramphenicol, and ciprofloxacin-resistant strain), Gram-positive multidrug-resistant strain of *S. aureus* (kanamycin, chloramphenicol, ciprofloxacin, ampicillin-resistant strain), and a yeast, *C. albicans*, were obtained from the San Vicente Hospital in Monterrey, Nuevo León, México. All the bacterial assays were performed in Müller-Hinton (MH) media (BD, Bioxon), and all the assays using *C. albicans* were performed in Yeast Malt (YM) media (BD, Bioxon).

### *Gardenia jasminoides* extract preparation

Leaves from the plant *G*. *jasminoides* were collected in Monterrey, Mexico. A voucher specimen (number 634) was deposited in ANSM Herbarium (Universidad Autónoma Agraria Antonio Narro) in Saltillo, Coahuila, México. Leaves were washed using deionized water to remove dust and were dried at room temperature (22–26 °C) for 24 h before being powdered. An aqueous extract was prepared as follows: two grams of powdered leaves were mixed with 60 mL of deionized water at room temperature, stirred for 2 h, and filtered immediately through Whatman filter paper. The aqueous extract of *G*. *jasminoides* was stored at 4 °C until their use in the synthesis of NPs.

### Synthesis of monometallic nanoparticles

A total of 12.5 mL of 0.02 M AgNO_3_ were mixed with 12.5 mL of *G. jasminoides* extract and heated for 1 h at 80 °C. The formation of silver nanoparticles was monitored through a change of color of the mixture (from transparent to brownish). The reaction was cooled and centrifuged at 14,000 rpm for 5 min. The product was then washed with 70% ethanol and lyophilized (FreeZone 6, Labconco, Kansas City, MO, USA) for 24 h to obtain the purified AgNPs. We followed the previous procedure for the synthesis of the iron nanoparticles; only 12.5 mL of 0.01 M Fe(NO_3_)_3_ solution was used instead of the silver solution, and the color change observed was from transparent to black.

### Synthesis of Ag–Fe bimetallic nanoparticles

The synthesis of Ag-Fe bimetallic nanoparticles was performed by adding 12.5 mL of 0.02 M AgNO_3_ and 12.5 mL of 0.01 M Fe(NO_3_)_3_ in a Falcon tube. Then, the tube was mixed and heated until reaching 60ºC. When this temperature was reached, 25 mL of the filtered *G. jasminoides* extract was added to the mixture (2:1:1 proportion), kept at constant stir, and heated at 80ºC for 1 h. The formation of the nanoparticles was monitored and confirmed by a change of color of the mix that changed from transparent to brown (for silver) or black (for iron). The reaction was cooled at room temperature, centrifuged at 14,000 rpm for 5 min, and the supernatant was discharged. The product was then washed with 70% ethanol, left to dry at room temperature for 24 h, and finally lyophilized (FreeZone 6, Labconco, Kansas City, MO, USA) for another 24 h.

### Nanoparticle characterization

Several techniques were used to characterize the synthesized nanoparticles, such as UV–visible (UV–vis) spectra, Fourier-transform infrared (FT-IR) spectroscopy, RAMAN spectroscopy, Transmission electron microscopy (TEM), High Resolution (HR)-TEM, dark-field microscopy, and Energy dispersive X-ray (EDX) analysis. UV–vis absorption spectra of the nanoparticles (1 mg/mL in distilled water as a dispersive medium) were studied in the 200–600 nm wavelength range using a Multiskan GO Microplate Spectrophotometer (Thermo Fisher Scientific, USA). FT-IR analysis was performed in the 650–4000 cm^−1^ range in a Shimadzu IRAffinity-1 spectrometer (Shimadzu, Japan) to determine the plant extract components and the synthesized nanoparticles. The lyophilized NPs and extract (1 mg/mL) were diluted in KBr and analyzed by the attenuated total reflectance method. To obtain information about the interactions between the nanoparticles and the plant extract, a RAMAN spectrum of the nanoparticles and the extract was measured using a DXR RAMAN microscope (Thermo Fisher Scientific, USA). The elemental, morphological, and size of the nanoparticles were obtained through a FEI-TITAN 80–300 kV scanning transmission electron microscope. Briefly, the sample was prepared by depositing and evaporating a drop of NPs solution (1 mg/mL) onto lacey carbon-coated copper grids. The elements presented in NPs were determined by an energy-dispersive spectrometry analyzer integrated in the transmission electron microscope. To analyze the particle size and size distribution, we used the TEM and HR-TEM micrographs to measure de diameter of NPs and then processed them with ImageJ software developed by the National Institutes of Health.

### Magnetic properties

A round-shaped magnet was used to produce an external magnetic field, having direct contact with the nanoparticles. If the nanoparticles adhered to the magnet, they were considered to exhibit magnetic properties.

### Determination of minimal inhibitory concentration (MIC)

The determination of MICs was performed by the microdilution method. Stocks of NPs (mono- and bimetallic) were prepared at a final concentration of 1000 ppm in LB or YM broth. To perform the MIC test, we used a 96-wells microtiter plaque (Corning, USA). We added in the top well the necessary volume from the stock solution to reach 500 ppm concentration of the NPs in a final volume of 200 μL. Then, serial dilutions were performed by taking 100 μL from every very next well into 100 μL of culture media. The last 100 μL from the last dilution was discarded. The concentration range was from 250 to 0.97 ppm of each NPs after the bacteria was added. For inoculation, an overnight (ON) culture was prepared at 37 °C, 150 rpm, and incubated for 20 h, Next, the ON culture was diluted 1:250 in fresh media and incubated until a critical optical density was reached (OD_600nm_ of 0.2 ± 0.02 for bacteria and an OD_600nm_ of 0.3 ± 0.02 cells/mL for yeast). Finally, a 1:100 dilution was made with fresh media, and 100 μL of this dilution was added to each test well. Plates were incubated at 37ºC 150 rpm for 20 h, and then, the OD_600nm_ was measured in a Multiskan GO Microplate Spectrophotometer (Thermo Fisher Scientific, USA). The MIC was defined as the minimal concentration at which no growth was observed. Each assay was performed in triplicates; growth and sterility controls were included.

The behavior of the bimetallic NPs against microbial growth was analyzed by the Bliss independence model as described by Hegreness et al.^56^, which indicates that synergy can be considered when the effect of the combined antimicrobial agent (bimetallic NP) is more significant than the predicted of its components (single metal NP). Therefore, Eq. 1 was used to analyze the synergistic properties of the bimetallic nanoparticles, where the *S* value; the difference between the predicted value of individual components *x* and *y* (*f*_*x0*_ and *f*_*0y*_, respectively) and the combined treatment *xy* (*f*_*xy*_) is denoted in the form:

1 $$S = \left( {\frac{{f_{x0} }}{{f_{00} }}} \right)\left( {\frac{{f_{0y} }}{{f_{00} }}} \right) - \frac{{f_{xy} }}{{f_{00} }}\quad {\text{Bliss}}\;{\text{Independence}}\;{\text{Model}}$$

where, $f_{x0}$ = treatment with Ag, $f_{0y}$ = treatment with the transition metal, $f_{xy}$ = treatment Ag + metal, $f_{00}$ = growth control of a culture that has not been treated with the transition metals. The value of S describes the interaction between combinatorial treatments as follows: S > 0 Synergistic; S = 0 Additive; S < 0 Antagonistic.

### Determination of minimum bactericidal and minimum fungicidal concentration (MBC, MFC)

After obtaining the monometallic and bimetallic nanoparticles MICs, the wells that corresponded to the twice of the MIC, the MIC, and the growth control of each treatment were selected. Serial dilutions of selected wells were performed until they reach a final concentration of 1 × 10^7^ cells/mL. Ten microliters of each dilution were cultured in a Petri dish, incubated at 37ºC for 24 h, and finally, counted the colony-forming units (CFU) in each treatment.
